# Provision of Digital Health Technologies for Opioid Use Disorder Treatment by US Health Care Organizations

**DOI:** 10.1001/jamanetworkopen.2023.23741

**Published:** 2023-07-17

**Authors:** Chris Miller-Rosales, Nancy E. Morden, Mary F. Brunette, Susan H. Busch, John B. Torous, Ellen R. Meara

**Affiliations:** 1 Department of Health Care Policy, Harvard Medical School, Boston, Massachusetts; 2 Dartmouth Institute for Health Policy and Clinical Practice, Geisel School of Medicine, Lebanon, New Hampshire; 3 UnitedHealthcare, Minnetonka, Minnesota; 4 Department of Psychiatry, Geisel School of Medicine, Dartmouth College, Hanover, New Hampshire; 5 Bureau of Mental Health Services, New Hampshire Department of Health and Human Services, Concord; 6 Department of Health Policy and Management, Yale School of Public Health, New Haven, Connecticut; 7 Department of Psychiatry, Beth Israel Deaconess Medical Center, Harvard Medical School, Boston, Massachusetts; 8 Department of Health Policy and Management, Harvard T.H. Chan School of Public Health, Boston, Massachusetts

## Abstract

**Importance:**

Digital health technologies may expand organizational capacity to treat opioid use disorder (OUD). However, it remains unclear whether these technologies serve as substitutes for or complements to traditional substance use disorder (SUD) treatment resources in health care organizations.

**Objective:**

To characterize the use of patient-facing digital health technologies for OUD by US organizations with accountable care organization (ACO) contracts.

**Design, Setting, and Participants:**

This cross-sectional study analyzed responses to the 2022 National Survey of Accountable Care Organizations (NSACO), collected between October 1, 2021, and June 30, 2022, from US organizations with Medicare and Medicaid ACO contracts. Data analysis was performed between December 15, 2022, and January 6, 2023.

**Exposures:**

Treatment resources for SUD (eg, an addiction medicine specialist, sufficient staff to treat SUD, medications for OUD, a specialty SUD treatment facility, a registry to identify patients with OUD, or a registry to track mental health for patients with OUD) and organizational characteristics (eg, organization type, Medicaid ACO contract).

**Main Outcomes and Measures:**

The main outcomes included survey-reported use of 3 categories of digital health technologies for OUD: remote mental health therapy and tracking, virtual peer recovery support programs, and digital recovery support for adjuvant cognitive behavior therapy (CBT). Statistical analysis was conducted using descriptive statistics and multivariable logistic regression models.

**Results:**

Overall, 276 of 505 organizations responded to the NSACO (54.7% response rate), with a total of 304 respondents. Of these, 161 (53.1%) were from a hospital or health system, 74 (24.2%) were from a physician- or medical group–led organization, and 23 (7.8%) were from a safety-net organization. One-third of respondents (101 [33.5%]) reported that their organization used at least 1 of the 3 digital health technology categories, including remote mental health therapy and tracking (80 [26.5%]), virtual peer recovery support programs (46 [15.1%]), and digital recovery support for adjuvant CBT (27 [9.0%]). In an adjusted analysis, organizations with an addiction medicine specialist (average marginal effect [SE], 32.3 [4.7] percentage points; *P* < .001) or a registry to track mental health (average marginal effect [SE], 27.2 [3.8] percentage points; *P* < .001) were more likely to use at least 1 category of technology compared with otherwise similar organizations lacking these capabilities.

**Conclusions and Relevance:**

In this cross-sectional study of 276 organizations with ACO contracts, organizations used patient-facing digital health technologies for OUD as complements to available SUD treatment capabilities rather than as substitutes for unavailable resources. Future studies should examine implementation facilitators to realize the potential of emerging technologies to support organizations facing health care practitioner shortages and other barriers to OUD treatment delivery.

## Key Points

**Question:**

How often are digital health technologies for opioid use disorder used by US health care organizations, and which organizational features are associated with their use?

**Findings:**

In this cross-sectional study using data from a national survey of 276 organizations with accountable care organization contracts, 34% used at least 1 category of technology, including remote mental health therapy and tracking (27%), virtual peer recovery support programs (15%), and digital recovery support for adjuvant cognitive behavior therapy (9%). Organizations with traditional substance use disorder resources were significantly more likely to use technologies.

**Meaning:**

These results suggest that health care organizations use digital health technologies as complements to, rather than substitutes for, traditional resources.

## Teaser

This cross-sectional study uses national survey data to examine patient-facing digital health technology use for opioid use disorder treatment by US organizations with accountable care organization (ACO) contracts.

## Introduction

Although estimates suggest that 2.5% of US residents have opioid use disorder (OUD), medication and behavioral treatment for OUD is scarce.^1,2,3^ Numerous barriers impede access to OUD treatment, including transportation barriers^2^ and insufficient numbers of mental health and substance use disorder (SUD) clinicians.^4,5,6,7^ Digital health technologies have the potential to mitigate such barriers and expand access to treatment for patients with OUD.^8,9,10^ However, the diffusion of digital health technologies could exacerbate disparities without careful attention to equity concerns, including understanding the clinical settings in which they are offered.^11,12,13^ Currently, scant information documents which emerging patient-facing digital health technologies for OUD are used by health care organizations and the types of organizations most likely to deploy them.

Three technology categories that can support OUD treatment include remote mental health therapy and tracking, virtual peer recovery support programs, and digital recovery support for adjuvant cognitive behavior therapy (CBT) for OUD. Remote mental health therapy and tracking includes telemedicine counseling and mobile resources to promote OUD self-management and treatment adherence. Mobile applications may support patients to continuously track patient-reported outcomes or reach a therapist for concerns related to pain and withdrawal symptoms. Research has demonstrated the feasibility and acceptability of remote mental health applications, although evidence on clinical effectiveness is limited.^14,15,16,17^ Virtual peer recovery support programs offer access to self-help groups to support long-term recovery. A recent Cochrane review suggested that 12-step programs supported abstinence from alcohol and may extend to support OUD recovery as well.^18^ Programs such as SMART Recovery (Self-Management and Recovery Training) provide remote services that can complement or extend professional treatment. Digital recovery support for adjuvant CBT includes technologies that can deliver behavioral interventions to support OUD treatment. For example, reSET-O is a software program with US Food and Drug Administration clearance for use as a medical device, with some evidence of improving adherence to treatment involving evidence-based medications for OUD (MOUD).^19,20^

It remains unclear whether health care organizations use emerging technologies for OUD as substitutes for or complements to available SUD treatment resources. Important SUD treatment resources include staffing to treat patients with SUD, MOUD prescribing capability, registries to track mental health and OUD, and close relationships with specialty SUD treatment facilities that may treat those with more severe illnesses. Organizations with substantial resources may have the ability to effectively integrate digital services. On one hand, organizations can extend treatment provided by their clinicians through mobile tools to track mental health symptoms remotely.^21^ On the other hand, technologies could substitute for insufficient SUD resources to meet clinical demand for patients with OUD. If technologies are primarily available in organizations with robust SUD treatment resources, then they are not yet reaching their full potential to advance access to care for patients with unmet needs in organizations without traditional treatment alternatives.

Value-based payment structures may promote the use of emerging technologies if they improve outcomes and reduce costs.^22^ Accountable care organizations (ACOs) are health care organizations in the US responsible for the quality and costs of care of a designated population. These organizations tend to be leaders in the adoption of care delivery innovations designed to meet population health goals while limiting costs; therefore, they are well positioned to implement digital health technologies for OUD.

Despite the potential for value-based care organizations to be digital health leaders and the fact that health care organizations are purchasers for the majority of digital health technologies,^23^ no study to date has characterized the use of patient-facing digital health technologies for OUD in organizations with ACO contracts. Using data from a national survey of ACOs, we examined 2 research questions: (1) How often are patient-facing digital health technologies for OUD deployed by ACOs in the US? and (2) Which organizational features are associated with the use of digital health technologies for OUD? Specifically, we examined whether organizations with greater SUD treatment resources were more likely to use digital health technologies for patients with OUD.

## Methods

### Study Design

We administered the 2022 National Survey of Accountable Care Organizations (NSACO) to all organizations participating in Medicare and Medicaid ACO contracts. Building on prior waves of the NSACO, the 2022 survey added questions related to SUD treatment resources. We sent paper and electronic surveys to leaders (population health officers, chief operating officers, and Medicare ACO public contacts) of 505 organizations between October 1, 2021, and June 30, 2022, yielding 304 respondents from 276 organizations (54.7% response rate). The eFigure in Supplement 1 summarizes survey administration, and eTable 1 in Supplement 1 displays the survey questions used. The study outreach, including informed consent, was approved by the Harvard University Institutional Review Board. This study followed the Strengthening the Reporting of Observational Studies in Epidemiology (STROBE) reporting guideline.

### Study Variables

#### Outcomes

Based on recommendations from authors and an external advisory board with subject matter expertise in mental health and SUD services, we identified 3 technology categories: remote mental health therapy and tracking, virtual peer recovery support programs, and digital recovery support for adjuvant CBT, as displayed in Table 1. We generated binary indicators of whether organizations used each individual category. We then generated a count of the total categories of digital health technologies used overall (range, 0-3) as well as a binary measure of whether the ACO offered at least 1 technology.

**Table 1.** Survey Items Measuring 3 Categories of Digital Health Technologies for Opioid Use Disorder (OUD) Included in the 2022 National Survey of Accountable Care Organizations

Variable	Survey wording	Response
Remote mental health therapy and tracking	Which of the following strategies, if any, do clinicians in your organization use to integrate treatment for OUD and mental illness? If provided directly and via referral, check both.	Yes, provide directly
		Yes, refer out
		No
		Descriptive analyses reported 4 response categories: the above 3 plus 1 (Yes, both provide directly and refer out)
	Digital therapy or other resources to track mental health symptoms and promote OUD self-management (eg, BetterHelp or similar digital tools for smartphone or computer)	For our multivariable regression model, we collapsed responses to reflect any use of remote mental health therapy and tracking, either provided directly or referred out (vs No)
Virtual peer recovery support programs	Do clinicians in your organization delivering services to patients with opioid use disorder provide any of the following services, either directly or via referral?	Yes
	Virtual recovery programs (eg, SMART Recovery or other virtual peer recovery groups accessed via mobile device or computer)	No
Digital recovery support for adjuvant cognitive behavior therapy	Do clinicians in your organization delivering services to patients with opioid use disorder provide any of the following services, either directly or via referral?	Yes
	Digital recovery support services (eg, reSET-O or similar prescription digital therapeutic intended to provide adjuvant cognitive behavioral therapy)	No

Abbreviation: SMART, Self-Management and Recovery Training.

#### SUD Treatment Resources

Respondents reported whether their organization included an addiction medicine specialist (yes or no) and whether they agreed that the organization had sufficient staff to treat the needs of patients with SUD (strongly agree or somewhat agree).

The gold-standard treatment for OUD is the prescription of MOUD, yet attitudinal, training, regulatory, and administrative barriers have limited MOUD availability.^24,25^ Until 2023, clinicians required additional training to prescribe buprenorphine, and methadone treatment has been limited to a small number of highly regulated certified programs. Consequently, ACOs may not include clinicians who are able to prescribe MOUD. We included a binary measure of whether the ACO had the capability to prescribe at least 1 MOUD (buprenorphine, naltrexone, or methadone [yes or no]).

We created a binary measure of whether the largest ACO contract participated with at least 1 specialty SUD treatment facility, including outpatient (eg, certified opioid treatment programs), inpatient, and residential treatment facilities (yes or no). Organizations reported whether they had a patient registry to identify patients with OUD or a registry to track mental health symptoms and treatment response, reported as binary outcomes. Beyond having functionality within the electronic health record to create registries, our survey asked whether clinicians in the organization routinely used registries or dashboards to facilitate treatment for patients with OUD.

#### ACO Characteristics

We included a categorical measure of organization type, including hospital or health system, physician or medical group led, safety-net organization (eg, Federally Qualified Health Center or coalition), or other organization. We included binary variables measuring the following: whether the organization included a Medicaid ACO contract; whether respondents agreed or strongly agreed that staffing, specialized training, and other costs can be a barrier to delivering mental health and SUD treatment services; and whether the ACO had a management partner (a third-party organization that could provide data, administrative, or educational services^26^). Last, we included a measure for the census region of the state where the ACO was based (Midwest, Northeast, South, or West) as well as a binary measure of whether the survey was taken on paper or online.

### Statistical Analysis

First, we described characteristics of the overall sample of ACOs; second, we used χ^2^ tests to test whether descriptive characteristics differed comparing organizations that used at least 1 category of technology vs organizations that used none. We then calculated the use of each category of digital health technology for OUD as well as the number of categories used.

We fit 4 separate models estimating the likelihood of reporting using the following: (1) at least 1 patient-facing technology category (vs none), (2) remote mental health therapy and tracking, (3) virtual peer recovery support programs, and (4) digital recovery support for adjuvant CBT. Each multivariable logistic regression model included variables to test whether ACOs with greater resources (included an addiction medicine specialist, reported sufficient staff to treat SUD, offered MOUD, included a specialty SUD treatment facility, had a registry to identify patients with OUD, or had a registry to track mental health for patients with OUD) were more likely to use digital health technologies for OUD compared with ACOs lacking these resources, after adjusting for organization type, inclusion of a Medicaid contract, financial barriers to treatment, having a management partner, census region, and survey type. Results are reported as average marginal effects, or the estimated change in the probability of reporting patient-facing technology use for a change in each independent variable, to improve interpretability and to enable comparisons with models based on different explanatory variables and study samples.^27,28^

We designated missing responses to the binary availability of resources as not available. As a sensitivity analysis, we conducted a complete case analysis, only estimating models based on respondents who completed all survey items. Because outreach included more than 1 respondent per organization and respondents were encouraged to share surveys with colleagues if they were uncertain about survey items, estimates accounted for 26 organizations with more than 1 respondent. For each organization, we created an analytic weight equal to 1/*k* responses, where *k* was the total number of responses per organization, and we clustered SEs at the organization level. We conducted all analyses including weights in the svy suite of commands in Stata, version 17.0 (StataCorp LLC). Data analysis was performed between December 15, 2022, and January 6, 2023.

## Results

### Descriptive Characteristics of ACO Respondents

Of the 276 organizations with an ACO contract, there were 304 respondents; 161 (53.1%) were from a hospital or health system (52.7%), 74 (24.2%) were from a physician- or medical group−led organization, and 23 (7.8%) were from a safety-net organization (Table 2). Specialty SUD resource availability varied widely, from sufficient staff to treat SUD reported by 40 respondents (12.6%) to having a registry to identify patients with OUD reported by 172 respondents (57.0%). Table 2 reports organizational characteristics overall and by the main outcome: presence of a digital health technology. Medicare Shared Savings Program data on structural, contract, and performance characteristics indicated that the respondent sample was similar along nearly all domains to nonrespondent peers in the Medicare Shared Savings Program (eTable 2 in Supplement 1).

**Table 2.** Unadjusted Characteristics of Accountable Care Organizations, Overall and by Users of Digital Health Technologies for Opioid Use Disorder (OUD)^a^

Characteristic	Overall sample (N = 304)	No. of digital health technology categories reported			
		None (n = 203 [66.5])	At least 1 (1-3) (n = 101 [33.5])	*P* value
SUD treatment resource				
Addiction medicine specialist	115 (37.4)	38 (18.7)	77 (74.5)	<.001
Sufficient staff to treat SUDs	40 (12.6)	16 (7.8)	24 (22.3)	<.001
Specialty SUD treatment facility	159 (52.6)	90 (45.1)	69 (67.4)	<.001
Medications for OUD	159 (52.0)	86 (41.8)	73 (72.3)	<.001
Registry to identify patients with OUD	172 (57.0)	95 (47.1)	77 (76.6)	<.001
Registry to track mental health for patients with OUD	84 (27.5)	23 (11.5)	61 (59.5)	<.001
Accountable care organization				
Organization type				
Hospital or health system	161 (53.1)	104 (51.6)	57 (56.0)	.01
Physician or medical group led	74 (24.2)	52 (25.7)	22 (21.7)	
Safety-net hospital	23 (7.8)	9 (4.5)	14 (14.4)	
Other (eg, payers)	48 (14.9)	39 (18.4)	9 (8.4)	
Includes Medicaid contract	154 (49.5)	91 (43.0)	63 (62.5)	.002
Reports financial barriers to treatment	164 (55.3)	95 (48.9)	69 (67.9)	.01
Management partnership	66 (21.7)	40 (20.5)	26 (24.2)	.48
Region				
South	72 (23.3)	50 (24.3)	22 (21.2)	.77
Midwest	67 (22.9)	45 (23.5)	22 (21.7)	
Northeast	110 (37.1)	70 (35.0)	40 (41.3)	
West	55 (16.7)	38 (17.2)	17 (15.8)	
Paper survey (vs online)	23 (6.6)	12 (5.1)	11 (9.5)	.15

Abbreviation: SUD, substance use disorder.

^a^ Unless indicated otherwise, values are presented as No. (%) of respondents. These data are from the 2022 National Survey of Accountable Care Organizations. Survey respondents participated in at least 1 Medicare or Medicaid accountable care organization contract. Differences between organizations that used at least 1 category of digital health technology for OUD vs organizations that used none were compared with χ^2^ tests. Analytic weights were applied to 26 organizations with more than 1 respondent so that each of the 276 unique organizations had equal weight in all estimates, and clustered SEs at the organization level accounted for correlation of responses within an organization. Unweighted frequencies and weighted percentages are reported.

### Prevalence of Digital Health Technologies for OUD

One-third of ACOs (101 [33.5%]) used at least 1 category of technology (Table 3). The most commonly used category was remote mental health therapy and tracking, reported by 80 respondents (26.5%). Remote mental therapy and tracking was more likely to occur via referral (57 [19.5%] overall) than direct provision (17 [5.6%] overall), while 6 respondents (1.4%) provided this technology both directly and referred externally. In total, 46 respondents (15.1%) used virtual peer recovery support programs and 27 (9.0%) used digital recovery support for adjuvant CBT.

**Table 3.** Overall Use of Digital Health Technologies for Opioid Use Disorder in Accountable Care Organizations^a^

	Overall use (N = 304)
Digital health technology	
Remote mental health therapy and tracking, any use	80 (26.5)
Provided directly	17 (5.6)
Provided both directly and referred out	6 (1.4)
Referred out	57 (19.5)
Virtual peer recovery support programs	46 (15.1)
Digital recovery support for adjuvant cognitive behavior therapy	27 (9.0)
No. of technology categories used (0-3)	
0	203 (66.5)
1	66 (22.2)
2	18 (5.5)
3	17 (5.8)
≥1	101 (33.5)

^a^ Unless indicated otherwise, values are presented as No. (%) of respondents. These data are from the 2022 National Survey of Accountable Care Organizations. Analytic weights were applied to 26 organizations with more than 1 respondent so that each of the 276 unique organizations had equal weight in all estimates, and clustered SEs at the organization level accounted for correlation of responses within an organization. Unweighted frequencies and weighted percentages are reported.

### Factors Associated With the Use of Technologies in Adjusted Models

After adjusting for all model covariates, ACOs with an addiction medicine specialist were 32.3 (SE, 4.7) percentage points (*P* < .001) more likely to use at least 1 category of technology compared with ACOs without an addiction medicine specialist (Table 4). Accountable care organizations that had a registry to track mental health were 27.2 (SE, 3.8) percentage points (*P* < .001) more likely to use at least 1 category of technology compared with ACOs that did not have a registry. Reports of sufficient staffing, a specialty SUD treatment facility, prescription of MOUD, and a registry to identify patients with OUD were not associated with technology use in an adjusted model. Physician- or medical group–led organizations were 13.2 (SE, 5.6) percentage points (*P* = .02) more likely to use at least 1 category of technology compared with hospital or health systems after adjusting for model covariates. Financial barriers, inclusion of a Medicaid contract, and management partnerships were not associated with technology use in an adjusted model.

**Table 4.** Association Between Substance Use Disorder (SUD) Treatment Resources and Adoption of Digital Health Technologies for Opioid Use Disorder (OUD) in Accountable Care Organizations^a^

Characteristic	Average marginal effect (SE)			
	Any technology category used (1-3 total)	Remote mental health therapy and tracking	Virtual peer recovery support programs	Digital recovery support for adjuvant CBT
SUD treatment resource				
Addiction medicine specialist	44.0 (7.6)^b^	47.2 (6.8)^b^	15.3 (5.0)^c^	4.3 (4.1)
Sufficient staff to treat SUDs	3.9 (7.6)	−1.2 (5.8)	9.6 (5.6)	4.7 (4.6)
Specialty SUD treatment facility	−0.6 (5.1)	−4.6 (4.7)	1.7 (4.2)	7.5 (3.6)^d^
Medications for OUD	−0.9 (5.3)	−6.8 (4.7)	2.4 (5.3)	4.2 (3.0)
Registry to identify patients with OUD	0 (4.7)	2.0 (5.0)	4.6 (4.8)	−1.4 (4.2)
Registry to track mental health for patients with OUD	36.3 (6.7)^b^	38.0 (6.6)^b^	7.7 (4.5)	8.9 (4.4)^d^
Accountable care organization				
Organization type				
Hospital or health system	[Reference]	[Reference]	[Reference]	[Reference]
Physician or medical group led	13.0 (5.5)^d^	9.8 (5.3)	4.8 (5.8)	13.4 (5.6)^c^
Safety-net hospital	13.2 (10.0)	−13.0 (5.1)^d^	11.4 (7.3)	11.5 (8.4)
Other	4.5 (6.5)	6.6 (6.3)	−6.7 (4.9)	−0.2 (4.9)
Includes Medicaid contract	1.8 (4.5)	3.8 (4.4)	2.0 (3.8)	−2.1 (3.8)
Reports financial barriers to treatment	−2.0 (4.3)	−4.4 (3.9)	9.9 (3.9)^c^	6.9 (3.3)^d^
Management partnership	4.9 (5.3)	6.6 (4.8)	3.1 (4.7)	−2.2 (3.7)
Region				
South	[Reference]	[Reference]	[Reference]	[Reference]
Midwest	−13.0 (5.2)^d^	−14.2 (4.8)^c^	−0.3 (4.9)	0.1 (4.7)
Northeast	−12.7 (5.9)^d^	−6.3 (5.2)	−6.5 (5.1)	−7.3 (4.0)
West	−15.6 (5.9)^c^	−8.2 (5.7)	−7.6 (4.7)	−6.6 (3.9)
Paper survey (vs online)	14.5 (9.5)	12.6 (9.1)	8.4 (6.5)	2.5 (6.5)

Abbreviation: CBT, cognitive behavior therapy.

^a^ These data are from the 2022 National Survey of Accountable Care Organizations. There were 304 respondents. Results are from 4 multivariable logistic regressions with separate outcomes: (1) whether the organization reported any of 3 digital health technology categories (1-3 used), (2) remote mental health therapy and tracking, (3) virtual peer recovery support programs, and (4) digital recovery support for adjuvant CBT. Average marginal effects were calculated to represent the expected average change in the probability of technology use, holding other variables at their observed values. Analytic weights were applied to 26 organizations with more than 1 respondent so that each of the 276 unique organizations had equal weight in all estimates, and clustered SEs at the organization level accounted for correlation of responses within an organization.

^b^ *P* < .001.

^c^ *P* < .01.

^d^ *P* < .05.

Adjusted models estimating the use of individual technology categories as outcomes demonstrated that ACOs that were a safety-net organization were 12.1 (SE, 5.2) percentage points (*P* = .02) less likely to use remote mental health therapy and tracking compared with hospital or health systems. After adjusting for model covariates, ACOs that agreed that costs could be a barrier to treatment were 10.7 (SE, 4.7) percentage points (*P* = .02) more likely to use virtual peer recovery support programs compared with ACOs without financial barriers. The results of sensitivity analyses using a complete case approach for missing responses estimated nearly identical associations, except the statistical significance of several variables was attenuated (eTable 3 in Supplement 1).

## Discussion

In this national cross-sectional study of 276 organizations with Medicare or Medicaid ACO contracts, we observed low and varied use of digital health technologies for OUD. Organizations with an addiction medicine specialist or a registry to track mental health were more likely to use at least 1 category of digital health technology for OUD compared with organizations lacking these resources. Although emerging technologies for OUD treatment represent a promising way to improve OUD service availability^4,5,6^ and thereby potentially advance equity in treatment access, our results suggest that their distribution, which was more prominent in organizations with other SUD treatment capabilities, is not yet addressing treatment access gaps.

Remote technologies can create or expand the availability of OUD services in health care organizations facing practitioner shortages and other limitations to OUD care delivery, but they can only achieve this if their uptake aligns with need. Our findings suggest a mismatch between need and deployment. Organizations with fewer SUD treatment resources were less likely to adopt emerging technologies. To address this mismatch, policy initiatives could focus efforts on overcoming barriers to technology implementation in high-need, resource-limited health care settings. For example, policy makers and payers might test policies and reimbursement schemes that support health care organizations without local SUD treatment resources to integrate digital health technologies for OUD into their practices and workflow. Initiatives to advance the uptake of technologies may address costs, knowledge, user engagement, organizational culture, leadership, interoperability, and data security concerns.^9,29,30,31,32,33,34,35,36,37,38,39^ Training and education for patients and clinicians may be a productive avenue to increase adoption.^36,40,41^ For example, Kaiser Permanente used both clinician referrals and direct-to-patient approaches to drive service use during a large-scale integration of digital mental health technologies.^42^ Future efforts may require investing in trained staff, such as digital navigators, to support patients and clinicians to overcome technological, workflow, and digital literacy constraints.^43,44^ Digital navigators offer an opportunity to overcome both patient- and staff-level barriers to technology use even in low-resource settings.

This study adds to an ongoing discussion of how digital health transformation might contribute to existing health disparities.^11,12,39,45,46,47^ Disparities in technology use may emerge through differential access for patients with limited health literacy and members of racial and ethnic minority groups, as documented during the diffusion of patient portal use^48,49^ and telehealth.^50,51,52^ Disparities may also emerge if uptake of innovations lags among organizations serving vulnerable patient populations, such as the challenges safety-net organizations have faced in the uptake of health information technology.^53,54,55,56,57^ Our unadjusted results showed that organizations serving vulnerable patient populations (safety-net organizations and organizations with a Medicaid ACO contract) were implementing digital health technologies; however, safety-net organizations were notably less likely to use remote mental health therapy and tracking compared with otherwise similar hospital and health systems. One possible explanation is that hospital and health system organizations have additional resources such as internal implementation assistance and greater technological infrastructure.^58^ Evidence on the effectiveness of emerging technologies among users of diverse racial, ethnic, and socioeconomic backgrounds may contribute to greater adoption among safety-net organizations and may make certain that the uptake of emerging technologies does not mirror existing disparities in access to SUD and mental health treatment.^59,60,61,62,63,64^

### Limitations

This study has important limitations. First, we reported statistical associations and cannot make causal inference. For example, we cannot identify direction or causality in our finding that ACOs reporting financial barriers to care were more likely to use virtual peer recovery support programs. Accountable care organizations might offer these programs because they face cost barriers, or they may face cost barriers due to offering these programs. Given the lack of research on current technology use, this descriptive analysis provides a foundation for developing causal hypotheses to test in future research. Second, we were unable to assess where organizations deployed technologies, how consistently local sites offered them, and individual patient uptake. For example, both primary care and specialist organizations can use technologies. Further, technologies may be both complements to and substitutes for SUD treatment resources, depending on heterogenous local site needs within large organizations. Future research should identify which health care organizations in the ACO network offer technologies. Third, our measure of specialty resources may overestimate patient access if they are not consistently available throughout the ACO—for example, if only a single clinician prescribes MOUD in the organization. Similarly, we were unable to measure characteristics of patients using the technologies for a more nuanced understanding of potential disparities. Fourth, we were unable to measure direct motivators for technology use or nonuse. For example, organizations may delay technology investment until there is greater evidence of their effectiveness through rigorous randomized studies or clearer identified local demand for their use. However, opioids currently contribute to most drug overdose deaths, and death rates due to overdose are high throughout the US (at least 18 per 100 000 in 46 states), suggesting that all organizations should build resources for patients with OUD.^65^ Fifth, we had a survey response rate of 54.7% and relied on individual respondents to measure organization-wide resources. Although we were unable to assess potential selection bias of respondents, available Medicare Shared Savings Program data on structural, contract, and performance characteristics indicate that the respondent sample was similar along nearly all domains to nonrespondent peers in the Medicare Shared Savings Program. Despite these limitations, this survey analysis provides important data on deployment of technologies for OUD in the context of ACOs, organizations that should have the capability to implement novel treatment practices to improve quality and access.

## Conclusions

Health care organizations are purchasers for the majority of digital health technologies,^23^ yet there is little research on their use of patient-facing digital health technologies for OUD at a national scale. In this cross-sectional study with national survey data, we measured the current use of digital health technologies for OUD in US organizations holding Medicare or Medicaid ACO contracts. Our results suggest that digital health technologies for OUD are more likely to be deployed by organizations with relatively robust traditional SUD treatment resources. As such, the technology appears to complement existing SUD treatment resources rather than substitute for unavailable SUD treatment resources. Future studies should examine implementation facilitators to realize the potential of digital health technologies to support organizations facing practitioner shortages and other barriers to OUD treatment delivery.

## Notes

Supplement 1. **eFigure.** Flowchart of the 2022 National Survey of Accountable Care Organizations **eTable 1.** Survey Questions for Variables Included in the Study Analysis **eTable 2.** Comparisons Between Characteristics of Respondents to the National Survey of Accountable Care Organizations and Nonrespondent Organizations in the Medicare Shared Savings Program **eTable 3.** The Association Between Substance Use Disorder Treatment Resources and Adoption of Digital Health Technologies for Opioid Use Disorder in Accountable Care Organizations

Supplement 2.
Data Sharing Statement
