# Supplementary material for: Provision of Digital Health Technologies for Opioid Use Disorder Treatment by US Health Care Organizations
Source: JAMA Netw Open. 2023 Jul 17;6(7):e2323741. doi: 10.1001/jamanetworkopen.2023.23741 (PMC10352858; doi:10.1001/jamanetworkopen.2023.23741)
Supplement: Supplement 1. — eFigure. Flowchart of the 2022 National Survey of Accountable Care Organizations eTable 1. Survey Questions for Variables Included in the Study Analysis eTable 2. Comparisons Between Characteristics of Respondents to the National Survey of Accountable Care Organizations and Nonrespondent Organizations in the Medicare Shared Savings Program eTable 3. The Association Between Substance Use Disorder Treatment Resources and Adoption of Digital Health Technologies for Opioid Use Disorder in Accountable Care Organizations [file jamanetwopen-e2323741-s001.pdf]

Supplemental Online Content

Miller-Rosales C, Morden NE, Brunette MF, Busch SH, Torous JB, Meara ER. Provision of digital health technologies for opioid use disorder treatment by US health care organizations. *JAMA Netw Open*. 2023;6(7):e2323741. doi:10.1001/jamanetworkopen.2023.23741

eFigure. Flowchart of the 2022 National Survey of Accountable Care Organizations

eTable 1. Survey Questions for Variables Included in the Study Analysis

eTable 2. Comparisons Between Characteristics of Respondents to the National Survey of Accountable Care Organizations and Nonrespondent Organizations in the Medicare Shared Savings Program

eTable 3. The Association Between Substance Use Disorder Treatment Resources and Adoption of Digital Health Technologies for Opioid Use Disorder in Accountable Care Organizations

This supplemental material has been provided by the authors to give readers additional information about their work.

© 2023 Miller-Rosales C et al. *JAMA Network Open*.

eFigure. Flowchart of the 2022 National Survey of Accountable Care Organizations

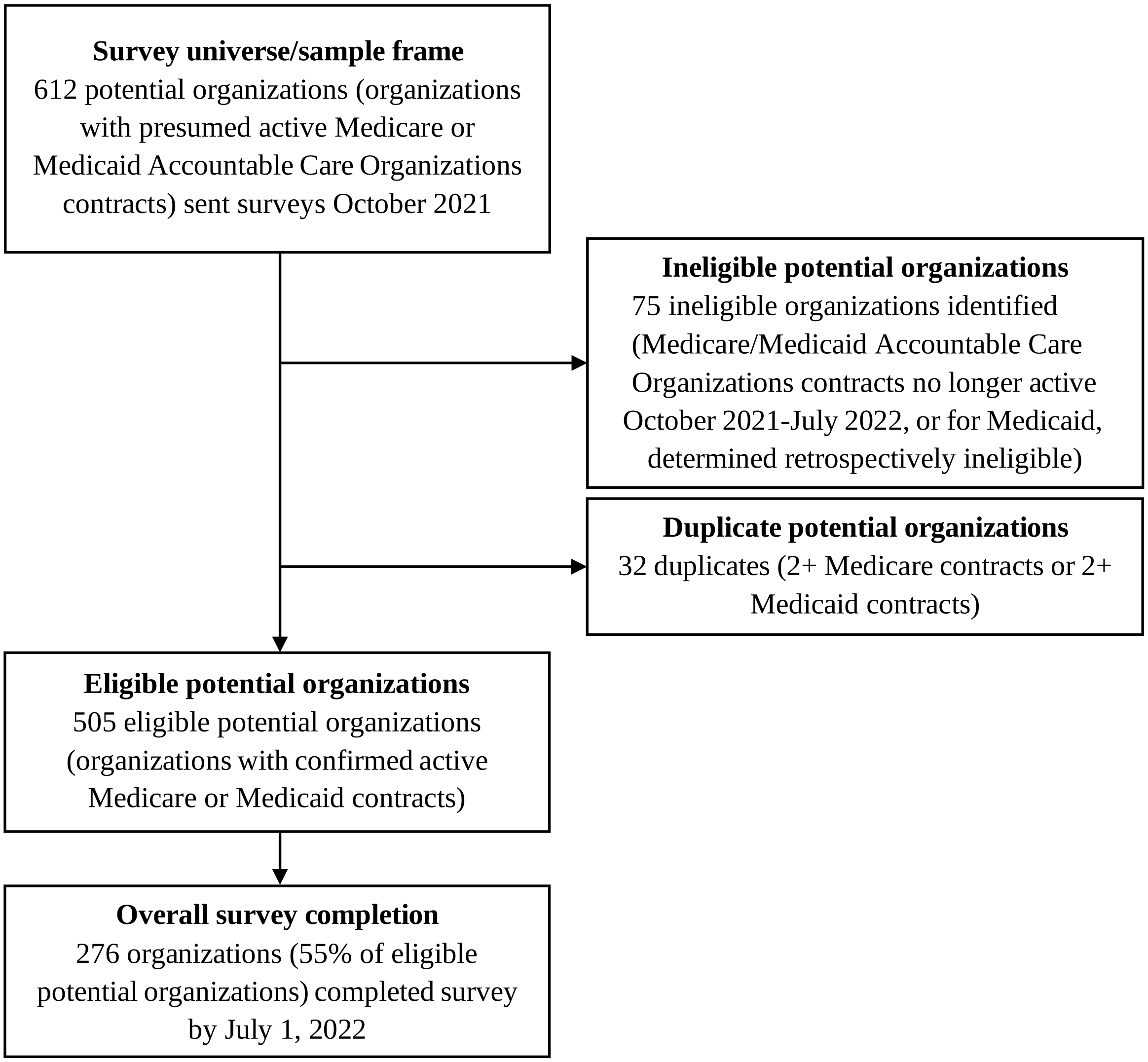

© 2023 Miller-Rosales C et al. *JAMA Network Open*.

eTable 1. Survey Questions for Variables Included in the Study Analysis

Variable	Survey wording	Responses
**Outcomes**		
Remote mental health therapy and tracking	Which of the following strategies, if any, do clinicians in your organization use to integrate treatment for OUD and mental illness? If provided directly and via referral, check both.<br><br>Digital therapy or other resources to track mental health symptoms and promote OUD self-management (e.g., BetterHelp or similar digital tools for smartphone or computer)	Yes, provide directly<br>Yes, refer out<br>No
Virtual peer recovery support programs	Do clinicians in your organization delivering services to patients with opioid use disorder provide any of the following services, either directly or via referral?<br><br>Virtual recovery programs (e.g., SMART Recovery or other virtual peer recovery groups accessed via mobile device or computer)	Yes<br>No
Digital recovery support for adjuvant cognitive behavioral therapy	Do clinicians in your organization delivering services to patients with opioid use disorder provide any of the following services, either directly or via referral?<br><br>Digital recovery support services (e.g., reSET-O or similar prescription digital therapeutic intended to provide adjuvant cognitive behavioral therapy)	Yes<br>No
**Substance use disorder treatment capabilities**		
Addiction medicine specialist	Does your organization include the following addiction treatment specialists?<br><br>Board-certified addiction psychiatry or addiction medicine specialist (i.e., psychiatrist or other physician certified to provide addiction treatment)	Yes<br>No
Sufficient staff to treat the needs of patients with substance use disorder	How strongly do you agree or disagree with this statement: *Our organization has sufficient staff to treat the needs of our patients with substance use disorder.*	Strongly agree<br>Somewhat agree<br>Neither agree nor disagree<br>Somewhat disagree<br>Strongly disagree
Medications for opioid use disorder	Do clinicians in your organization offer patients buprenorphine (i.e., Subutex, Suboxone) for OUD?<br><br>Do clinicians in your organization offer patients methadone for OUD?<br><br>Do clinicians in your organization offer patients naltrexone (i.e., Revia, Vivitrol) for OUD?	Yes<br>No

© 2023 Miller-Rosales C et al. *JAMA Network Open*.

Specialty substance use disorder treatment facility	We'd like to know whether your largest ACO contract (as measured by total attributed members) includes any specialty substance use treatment providers. Please identify whether the following facilities are participating in your largest ACO contract:<br><br><u>Outpatient Services</u><br>SAMHSA certified narcotic treatment program that provides methadone (Opioid Treatment Program (OTP))<br>Other outpatient substance use treatment program (Not SAMHSA certified narcotic treatment program/OTP)<br>Bridge clinic, or similar addiction clinic that provides substance use treatment for patients recently discharged from hospital or ED who are not yet connected to residential or other outpatient substance use treatment<br><br><u>Inpatient</u><br>Inpatient substance use treatment facility<br><br><u>Residential</u><br>Residential substance use treatment facility	Yes<br>No
Patient registry to identify patients with opioid use disorder	We are interested in how clinicians in your organization identify patients with Opioid Use Disorder (OUD). How often do clinicians in your organization use the following strategies to identify patients with OUD?<br><br>Patient dashboard or registry	Never<br>Sometimes<br>Most of the time<br>All of the time
Patient registry to track mental health symptoms	Do clinicians in your organization use the following strategies to identify and monitor mental illness among your patients with OUD?<br><br>Patient registry to track mental health symptoms and treatment response	
**Accountable Care Organization characteristics**		
Organization type	Is your organization a:<br>Hospital<br>Health system (includes hospital(s) and medical practices)<br>Medical group (includes medical practices)<br>Safety-net provider (e.g., FQHC or coalition)<br>Other (fill in)	One choice
Medicaid Accountable Care Organization contract	Do you currently have an ACO or ACO-like contract directly with a state Medicaid program (e.g., with a public Medicaid agency, as opposed to a managed care organization)?	Yes<br>No

© 2023 Miller-Rosales C et al. *JAMA Network Open*.

	Do you currently have an ACO contract with a private health plan (e.g., managed care organization) serving Medicaid enrollees?	
Financial barriers to treatment	Fixed costs can be a barrier to providing substance use treatment and mental health services. How strongly do you agree or disagree with the following statement:<br><br>*Staffing, specialized training, and other costs can be a barrier to delivering mental health and substance use treatment services.*	Strongly agree<br>Somewhat agree<br>Neither agree nor disagree<br>Somewhat disagree<br>Strongly disagree
Third-party management partner	Is your ACO affiliated with a third-party administrator or external, non-provider partner organization (e.g., management services organization or administrative services partner)?	Yes<br>No

© 2023 Miller-Rosales C et al. *JAMA Network Open*.

eTable 2. Comparisons Between Characteristics of Respondents to the National Survey of Accountable Care Organizations and Nonrespondent Organizations in the Medicare Shared Savings Program

	Not Responded	Responded	p-value
**N**	247	227	
**Contract Characteristics (%)**			
2-sided risk (vs. 1 sided)	38.9%	43.4%	0.31
High-revenue (vs. low-revenue)	47.4%	49.6%	0.63
Participates in ACO Investment Model	1.6%	3.1%	0.29
Participates in Advanced Pay	1.2%	3.1%	0.16
Service Area (%)			
-Northeast	27.5%	25.4%	0.61
-Midwest	26.7%	27.2%	0.91
-South	65.2%	57.0%	0.07
-West	15.0%	16.7%	0.62
-Multi-Region (not mutually exclusive of list above)	28.7%	22.4%	0.11
State with Medicaid Accountable Care Organizations	26.7%	18.9%	0.04
**Attributed Beneficiary Characteristics (2021)**			
N (mean)	21810	19618	0.28
Size Quartile (number beneficiaries)			
-1st quartile (2938-8205)	27.5%	22.4%	0.20
-2nd quartile (7261-12991)	22.3%	28.1%	0.15
-3rd quartile (13136-23732)	23.9%	26.3%	0.54
-4th quartile (23853-214009)	26.3%	23.2%	0.44
Dual-Eligible (%)	13	13	0.47
Disabled (%)	9.5%	10.4%	0.11
Race/Ethnicity (%)			
-White	87.4%	86.8%	0.64
-Black	7.9%	8.2%	0.65
-Asian	2.0%	1.9%	0.95
-Native	0.2%	0.2%	0.34
-Other	3.7%	4.0%	0.22
-Hispanic	1.7%	1.6%	0.88
Age (%)			
-Under 65	11.8%	12.8%	0.09
-65-74	48.2%	48.3%	0.81
-75-84	30.5%	29.8%	0.03
-85 plus	12.3%	11.9%	0.29

© 2023 Miller-Rosales C et al. *JAMA Network Open*.

**Providers in Contract**			
Any Federally Qualified Health Centers	23.5%	23.7%	0.96
Any Critical Access Hospitals	22.3%	18.0%	0.25
Any Rural Health Clinics	34.8%	31.1%	0.40
Any Elected Teaching Amendment Hospitals	0.0%	0.4%	0.30
Primary Care Providers (mean number)	320	281	0.32
Specialists (mean number)	616	531	0.35
**Utilization Measures**			
Inpatient discharges per 1000 beneficiary-years	262	265	0.51
Psych Discharges per 1000 beneficiary-years	5	5	0.76
Emergency department visits per 1000 beneficiary-years (outpatient)	610	608	0.85
Emergency department visits per 1000 beneficiary-years (inpatient)	191	194	0.55
Primary Care services per 1000 beneficiary-years	11013	10933	0.71
**Performance Measures**			
Consumer Assessment of Healthcare Providers and Systems (CAHPS)			
-CAHPS: Getting timely care	84.6%	84.8%	0.55
-CAHPS: How well your providers communicate	93.4%	93.7%	0.02
-CAHPS: Patients Rating of Provider	92.0%	92.4%	0.01
-CAHPS: Access to Specialists	78.7%	78.9%	0.51
-CAHPS: Health Promotion and Education	61.4%	61.8%	0.35
-CAHPS: Shared Decision Making	60.6%	61.2%	0.10
-CAHPS: Health Status/Functional Status	71.8%	71.8%	0.87
-CAHPS: Stewardship of Patient Resources	24.6%	24.8%	0.72
-CAHPS: Courteous and Helpful Office Staff	91.9%	91.9%	0.92
-CAHPS: Care Coordination	85.5%	85.8%	0.24
Percent of beneficiaries with hospital 30-day readmission	0.2%	0.2%	0.98
Rate of risk-adjusted all-cause Unplanned Admissions for patients with multiple chronic conditions	33.9	34.1	0.58
Percent of beneficiaries screened for future fall risk	87.3%	86.8%	0.66
Percent of beneficiaries who received a flu shot	80.8%	80.2%	0.50
Percent of beneficiaries screened for tobacco use and received cessation intervention if identified as a user	81.5%	80.4%	0.46
Percent of beneficiaries 12+ screened for depression and received follow-up plan if positive (Web Interface)	73.9%	74.9%	0.51
Percent of beneficiaries 12+ screened for depression and received follow-up plan if positive (electronic clinical quality measures)	43.2%	62.7%	0.12
Percent of beneficiaries 50-75 screened for colorectal cancer	73.8%	73.4%	0.73
Percent of female beneficiaries 50-74 screened for breast cancer (mammogram) in last 27 months prior to measurement period	75.3%	74.9%	0.66

© 2023 Miller-Rosales C et al. *JAMA Network Open*.

Percent of beneficiaries at high risk for cardiovascular disease event who were prescribed statin therapy	84.1%	84.4%	0.62
Percent of beneficiaries 12+ with dx of depression who reached remission at 12 months	15.9%	15.1%	0.52
Percent of patients 18-75 with diabetes who had a Hemoglobin A1C>9% (Web Interface)	12.6%	12.3%	0.68
Percent of patients 18-75 with diabetes who had a Hemoglobin A1C >9% (electronic clinical quality measures)	40.2%	38.2%	0.84
Percent of patients 18-85 with dx of hypertension who had blood pressure that was adequately controlled (Web Interface)	74.9%	74.9%	0.99
Percent of patients 18-85 with dx of hypertension who had blood pressure that was adequately controlled (electronic clinical quality measures)	65.0%	73.3%	0.06
Time in Medicare Shared Savings Program Contract (years)	5.5	5.3	0.47

Source: Medicare Shared Savings Program (MSSP) Accountable Care Organization Participant list, MSSP 2021 Performance Year Financial and Quality Results. P-values come from f-tests value in a linear model predicting response status (1=responded, 0=not responded) using the independent characteristic of interest.

© 2023 Miller-Rosales C et al. *JAMA Network Open*.

eTable 3. The Association Between Substance Use Disorder Treatment Resources and Adoption of Digital Health Technologies for Opioid Use Disorder in Accountable Care Organizations

	Any technology category used (1-3 total)		Remote mental health therapy and tracking		Virtual peer recovery support programs		Digital recovery support for adjuvant cognitive behavioral therapy	
	Average marginal effect (standard error)							
**Substance use disorder treatment resources**								
Addiction medicine specialist	39.6***	(8.5)	38.9***	(8.2)	22.8**	(7.3)	9.3	(6.7)
Sufficient staff to treat substance use disorders	8.6	(8.9)	1.6	(7.1)	14.7	(8.4)	4.5	(6.1)
Specialty substance use disorder treatment facility	1.2	(7.6)	-5.2	(7.2)	4.6	(6.6)	8.7	(5.8)
Medications for opioid use disorder	-2.0	(7.6)	-3.6	(7.0)	-1.0	(8.5)	5.8	(4.9)
Registry to identify patients with opioid use disorder	-5.1	(6.9)	0.3	(7.3)	6.7	(7.5)	-3.6	(7.3)
Registry to track mental health for patients with opioid use disorder	32.2***	(7.3)	33.2***	(7.1)	9.3	(6.9)	13.6*	(6.3)
**Accountable Care Organization characteristics**								
Organization type								
Hospital or health system	Reference organization type							
Physician/medical group-led	14.5	(7.5)	13.8	(7.3)	4.5	(9.3)	17.9*	(8.3)
Safety-net provider	9.1	(11.6)	-20.1**	(6.9)	17.9	(11.7)	6.8	(9.1)
Other	11.2	(9.2)	14.7	(9.3)	-8.3	(9.1)	1.3	(9.6)
Includes Medicaid contract	2.5	(7.2)	5.8	(6.8)	-0.2	(6.4)	-0.9	(6.3)
Reports financial barriers to treatment	-27.7**	(8.9)	-33.2***	(9.2)	15.1*	(6.5)	13.4**	(4.3)
Management partnership	12.7	(7.5)	13.3	(7.3)	1.4	(7.4)	0.5	(6.4)
**Region**								
South	Reference region							
Midwest	-8.6	(7.9)	-14.5	(7.6)	1.5	(7.6)	0.9	(7.2)
Northeast	-4.9	(8.9)	-1.6	(8.0)	-6.1	(8.6)	-6.9	(7.0)
West	-20.6*	(8.1)	-14.4	(8.0)	-8.2	(7.6)	-7.7	(6.2)
Paper survey (vs. online)	9.9	(9.7)	9.2	(10.6)	14.0	(9.6)	3.2	(10.1)
Observations	180							

Source: 2022 National Survey of Accountable Care Organizations. Results from four multivariable logistic regressions with separate outcomes: whether the organization reported any of three categories of digital health technologies (1-3 used), remote mental health therapy and tracking, virtual peer recovery support programs, and digital recovery support for adjuvant cognitive behavioral therapy. Average marginal effects were calculated to represent the expected average change in the probability of technology use, holding other variables at their observed values. Analytic weights were applied to 26 organizations with more than one respondent so that each organization had equal weight in all estimates, and clustered standard errors at the organization level accounted for correlation of responses within an organization. ***P<0.001; **P<0.01; *P<0.05

© 2023 Miller-Rosales C et al. *JAMA Network Open*.
