# Supplementary material for: Provision of Digital Health Technologies for Opioid Use Disorder Treatment by US Health Care Organizations
Source: JAMA Netw Open. 2023 Jul 17;6(7):e2323741. doi: 10.1001/jamanetworkopen.2023.23741 (PMC10352858; doi:10.1001/jamanetworkopen.2023.23741)
Supplement: Supplement 2. — Data Sharing Statement [file jamanetwopen-e2323741-s002.pdf]

Data Sharing Statement

Miller-Rosales. Provision of Digital Health Technologies for Opioid Use Disorder Treatment by US Health Care Organizations. *JAMA Netw Open*. Published July 17, 2023. doi:10.1001/jamanetworkopen.2023.23741

Data
Data available: No

Additional Information
Explanation for why data not available: The datasets generated during the current study are not publicly available due to privacy considerations of our survey respondents, but are available from the corresponding author on reasonable request.
